# Divergence of flowering-related genes to control flowering in five Euphorbiaceae genomes

**DOI:** 10.3389/fpls.2022.1015114

**Published:** 2022-10-19

**Authors:** Lan Jiang, Tingting Fan, Lihu Wang, Lin Zhang, Jun Xu

**Affiliations:** ^1^ Key Laboratory of Non-coding RNA Transformation Research of Anhui Higher Education Institution, Yijishan Hospital of Wannan Medical College, Wuhu, China; ^2^ Anhui Provincial Clinical Research Center for Critical Respiratory Disease, Wuhu, China; ^3^ Forestry College, Central South University of Forestry and Technology, Changsha, China; ^4^ School of Landscape and Ecological Engineering, Hebei University of Engineering, Handan, China; ^5^ College of Basic Medical Sciences, Hubei University of Chinese Medicine, Wuhan, China; ^6^ Hunan Institute of Microbiology, Changsha, China

**Keywords:** Euphorbiaceae, flowering-related genes, pathway, mechanisms, family

## Abstract

Reproductive growth and vegetative growth are a pair of main contradictions in the process of plant growth. Flowering, as part of reproductive growth, is a key switch in the life cycle of higher plants, which affects the yield and economic benefits of plants to a certain extent. The Euphorbiaceae species, including castor bean (*Ricinus communis*), physic nut (*Jatropha curcas*), tung tree (*Vernicia fordii*), cassava (*Manihot esculenta*), and rubber tree (*Hevea brasiliensis*), have important economic values because they are raw materials for the production of biodiesel, rubber, etc. The flowering mechanisms are still excluded in the Euphorbiaceae species. The flowering-related genes of *Arabidopsis thaliana* (Arabidopsis) were used as a reference to determine the orthologs of these genes in Euphorbiaceae genomes. The result showed that 146, 144, 114, 114, and 149 of 207 A*. thaliana* genes were respectively matched to *R. communis*, *V. fordii*, *J. curcas*, *H. brasiliensis*, and *M. esculenta*. These identified genes were clustered into seven pathways including gibberellins, floral meristem identity (FMI), vernalization, photoperiod, floral pathway integrators (FPIs), and autonomous pathways. Then, some key numbers of flowering-related genes are widely conserved in the Euphorbiaceae genomes including but not limited to FPI genes *LFY*, *SOC1*, *FT*, and FMI genes *AG*, *CAL*, and *FUL*. However, some genes, including *FRI*, *FLC*, and *GO*, were missing in several or all five Euphorbiaceae species. In this study, we proposed the putative mechanisms of flowering-related genes to control flowering and provided new candidate flowering genes for using marker-assisted breeding to improve variety quality.

## Introduction

Flowering is a key switch in the high plant life cycle. The developmental transition from vegetative growth to reproductive growth is regulated by multiple signaling pathways (Simpson and Dean, 2002). No matter when seeds and fruits are harvested, flowering is a premise for crop production in agriculture or forestry (Bluemel et al., 2015). The regulation of flowering time plays a very important role in the adaptation of crops to specific growth regions, so flowering time is a key topic of primary importance in agriculture or forestry. The identification and understanding of the function and structure of flowering-related genes may lay the foundation for further use of molecular-assisted breeding to cultivate new crop varieties with altered flowering times (Kim A. M. et al., 2013; Liu Y. et al., 2020). The introduction of early flowering-related genes may allow multiple rounds of cropping in single seasons or short growing seasons (Jung et al., 2012; Peng et al., 2015; Liu Y. et al., 2020). Additionally, transfer of genes that participated in late flowering may help increase the yield of crops by extending the time of vegetative growth (Putterill et al., 2004; Liu Y. et al., 2020).

For understanding the mechanism of plant flowering, many researchers have made some important progress on the molecular basis of flowering (Hecht et al., 2005; Mouhu et al., 2009; Jung et al., 2012; Peng et al., 2015; Liu Y. et al., 2020). In *Arabidopsis thaliana* (Arabidopsis), Fornara have isolated some mutants with loss of function and then identified more than 180 genes involved in regulating flowering time (Fornara et al., 2010). A result of the genetic analysis of *A. thaliana* mutants controlling flowering time suggested that the process of flowering involved a complex cross talk between different pathways responding to endogenous factors and environmental signals. The flowering transition process is mainly controlled by environmental signals (such as inter temperature (vernalization) and day length (photoperiod)) to ensure timely flowering (Bernier and Périlleux, 2005). Additionally, flowering time may be affected by ambient temperature, but the molecular mechanism of this pathway is still in the preliminary research stage (Lee et al., 2008; Cho et al., 2017). In addition to these external factors, the researchers also found that four floral pathways are closely related to flowering time in *A. thaliana*: GA (gibberellin) pathway, including autonomous pathway, vernalization response pathway, and photoperiod response pathway (Sheldon et al., 2000; Searle and Coupland, 2004; Simpson, 2004; Trevaskis et al., 2007; Jackson, 2009; Mutasa-Göttgens and Hedden, 2009). Compared to the wild type, mutations in genes such as *FPA*, *FVE*, *LD*, and *FCA* that participated in the autonomous pathway led to flowering under both short days and long days (Koornneef et al., 1991; Marquardt et al., 2006). On the contrary, mutations in genes participated in the long-day pathway, such as *FT*, *GI*, and *CO*, resulting in later flowering in long days but no delay in flowering under short days in comparison to the wild type (Koornneef et al., 1991; Cheng and Wang, 2005). Recently, in the control of vernalization and the circadian clock, the flowering mechanism has made great progress. The *FLC* containing a MADS-box domain seems to act as a flowering suppressor in *A. thaliana*, and its level is reduced after vernalization (Michaels and Amasino, 1999). The change of DNA methylation status was caused by the vernalization, which led to the repression of *FLC* expression (Bastow et al., 2004; Jean Finnegan et al., 2005). *ZTL* and *FKF1*, belonging to circadian clock-related genes, act as a bridge between circadian clock control and photoperiodic light signaling (Hoecker, 2005; Baudry et al., 2010). Researchers have confirmed that many flowering-related genes are widely conserved in the plant genomes. Putterill reported that about 85% of *A. thaliana* genes exist in other plant genomes; flowering-related genes have been identified and isolated from *Acacia mangium*, *Lotus corniculatus*, *Medicago truncatula*, *Glycine max*, and carnation (Putterill et al., 2004). Using genes related to flowering time from *A. thaliana* to detect the flowering-related genes in other plant genomes is an effective way (Hecht et al., 2005; Mouhu et al., 2009; Jung et al., 2012; Peng et al., 2015; Liu Y. et al., 2020), and these data provide resources for us to further understand the flowering time control beyond *A. thaliana*.

Bolting and flowering are the most important key life-history traits in the plant life cycle, which exercise far-reaching influence on evolution, gene flow, reproductive suitability, mating opportunities, and patterns (Post et al., 2008; Jung et al., 2016). The flowering strategies of plants show great diversity under different habitats and environmental conditions. Drought stress affects flowering, and this process was reported to promote flowering in *Sapium sebiferum*, *Citrus latifolia*, and *A. thaliana* (Southwick and Davenport, 1986; Riboni et al., 2013; Yang et al., 2015). The *GIGANTEA* (*GI*) gene, which is a repressor of FT from the photoperiod pathway, can accelerate flowering by suppressing *CYCLING DOF FACTOR* (*CDF*) or by binding to the *FT* promoter (Sawa and Kay, 2011). In addition, GI genes respond to conditions under salt, drought, and cold stresses, thereby helping plants adapt to unfavorable environments (Kim W.-Y. et al., 2013; Riboni et al., 2013; Fornara et al., 2015). The life cycles of plants can be adjusted systematically to adapt to different climates and latitudes. The model specie *A. thaliana*, which is an annual plant, can respond to vernalization and long day lengths. The Euphorbiaceae species castor bean (*Ricinus communis*) is an annual plant while tung tree (*Vernicia fordii*), physic nut (*Jatropha curcas*), cassava (*Manihot esculenta*), and rubber tree (*Hevea brasiliensis*) are perennials. To further elucidate the molecular mechanism of flowering in Euphorbiaceae, a large number of researchers have conducted limited studies. In contrast, most of the studies were carried out in the model plant *A. thaliana*. In *J. curcas*, RNA-seq analysis and molecular biology experiments have been carried out to identify and confirm genes controlling floral organ development and flowering time (Brasileiro et al., 2012; Li et al., 2014). In *V. fordii*, phenological, morphological, and histological experiments of tung flowers were conducted to give a comprehensive study of the flower biology and ontogeny (Li et al., 2020). Recently, the drafts of the *R. communis*, *V. fordii*, *J. curcas*, *M. esculenta*, and *H. brasiliensis* genome sequences were sequenced and reported (Chan et al., 2010; Wang W. et al., 2014; Ha et al., 2019; Zhang et al., 2019b; Liu J. et al., 2020). This genomic information lays a strong foundation for the genome-wide comparison of flowering-related genes between these Euphorbiaceae species and *A. thaliana*. The purpose of our study is to detect gene homologs associated with flowering among five Euphorbiaceae species by searching these whole-genome sequences. The divergence of Euphorbiaceae species *R. communis*, *V. fordii*, *J. curcas*, *M. esculenta*, and *H. brasiliensis* in genome duplication causes them to have different genome complexities and sizes (Chan et al., 2010; Wang W. et al., 2014; Ha et al., 2019; Zhang et al., 2019b; Liu J. et al., 2020). From this perspective, both of the differences among homologous genes and the distribution of the homologs were highlighted among these five Euphorbiaceae genomes. The results of this study obtained a large amount of gene resources, which provided a solid material basis for understanding the flowering mechanism of Euphorbiaceae and also provided a certain reference of other species.

## Materials and methods

### Data retrieval

Firstly, we obtained the genomes of *J. curcas*, *M. esculenta*, and *R. communi* from Phytozome and downloaded the genomes of *V. fordii* and *H. brasiliensis* from NCBI. Subsequently, a total of 207 A*. thaliana* genes involved in the flowering pathway as query sequences were obtained from the published paper and downloaded from TAIR. According to the HMM models and BlastP software, the flowering-related genes were identified in five Euphorbiaceae species. The obtained flowering-related genes were further confirmed by searching against NCBI (https://blast.ncbi.nlm.nih.gov/Blast.cgi) with non-redundant protein sequences.

### Domain analysis

We further analyzed all obtained flowering-related proteins with InterProScan using default parameters (Jones et al., 2014). In our study, we only chose the longest sequence from alternatively spliced transcripts for further analysis.

### Orthologous analysis and evolutionary tree constructe

To identify the orthologs between five Euphorbiaceae and *A. thaliana*, we carried out a collinear analysis using MCScanX (Wang et al., 2012). Firstly, we generated two files: proteins file and GFF file. Then, the proteins file was used to carry out a BlastP analysis with E-value 10-5. Finally, we plotted a collinear diagram by loading the BlastP file and GFF file. An evolutionary tree was constructed between five Euphorbiaceae and *A. thaliana* according to the methods of previous studies (Cao et al., 2020).

## Results and discussion

### Identification of flowering-related genes in Euphorbiaceae

**Figure 1:**
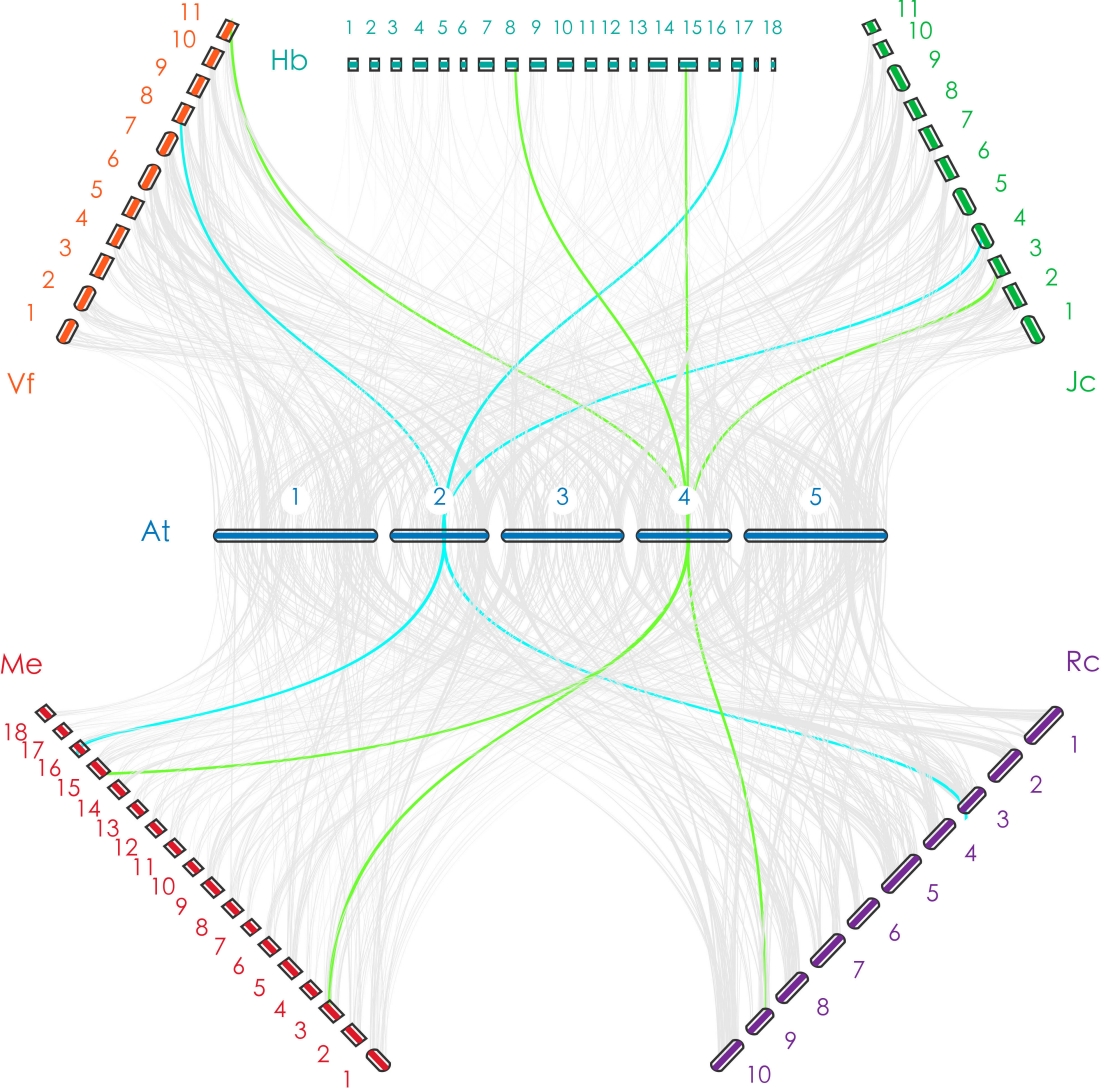
The orthologous relationship between *Arabidopsis thaliana* and Euphorbiaceae. The blue line indicates a one-to-one orthologous relationship (take ELF3 as an example); the green line indicates a one-to-one or one-to-two orthologous relationship (take ESD4 as an example).

To predict the orthologs of corresponding flowering-related genes of *A. thaliana* in the five Euphorbiaceae genomes, this review used 207 A*. thaliana* genes involved in the flowering pathway as query sequences. Among these genes, Jung (Jung et al., 2012) used an ortholog-based method to detect 24 genes with regulating flowering time, and Fornara (Fornara et al., 2010) have confirmed that 183 genes are involved in flowering regulatory pathways in a previous study. These genes for flowering pathways are mainly involved in the photoperiod pathway, the vernalization pathway, the gibberellin (GA) pathway, and the autonomous pathway, along with genes for floral meristem identity and floral pathway integrators (FPIs). In general, a gene can contain multiple functions involving different pathways; each gene was assigned to a single pathway according to its main function. Based on the HMM models, MCScanX, and BlastP software, the orthologs of *A. thaliana* flowering-related genes were identified in five Euphorbiaceae species (**Figure 1**), as described in several published papers (Cao et al., 2019a; Cao et al., 2019b; Cao et al., 2020). Finally, we found that 146, 144, 114, 114, and 149 of 207 A*. thaliana* genes were respectively matched to *R. communis*, *V. fordii*, *J. curcas*, *H. brasiliensis*, and *M. esculenta* genes and the numbers of these orthologous genes in each Euphorbiaceae were 176, 169, 130, 171, and 257, respectively (**Table 1**, **Table S1**, and **Table S2**).

**Table 1:** The number of orthologous genes of Arabidopsis thaliana flowering-related genes in the Ricinus communis, Jatropha curcas, Vernicia fordii, Manihot esculenta, and Hevea brasiliensis genomes.

			Species			
Pathway	*A. thaliana*	*R. communis*	*V. fordii*	*J. curcas*	*H. brasiliensis*	*M. esculenta*
No. of genes	207	176 (146)	169 (144)	130 (114)	171 (114)	257 (149)
Photoperiod	65	52 (43)	56 (47)	47 (39)	53 (34)	82 (46)
Vernalization	32	19 (15)	18 (15)	12 (11)	15 (11)	20 (15)
Autonomous	19	23 (18)	22 (18)	21 (17)	24 (13)	30 (19)
Floral pathway integrator	29	28 (22)	20 (18)	17 (17)	24 (19)	36 (23)
Ambient temperature	9	9 (7)	8 (6)	7 (5)	9 (6)	12 (7)
Gibberellin	2	2 (2)	2 (2)	2 (2)	1 (1)	5 (2)
Floral meristem identity	25	19 (16)	21 (17)	11 (10)	17 (11)	35 (17)
Unclassified	33	30 (27)	28 (25)	19 (18)	33 (23)	44 (25)

The numbers in parentheses indicate the number of A. thaliana genes with orthologous counterparts in each Euphorbiaceae.

### Photoperiod pathway

**Figure 2:**
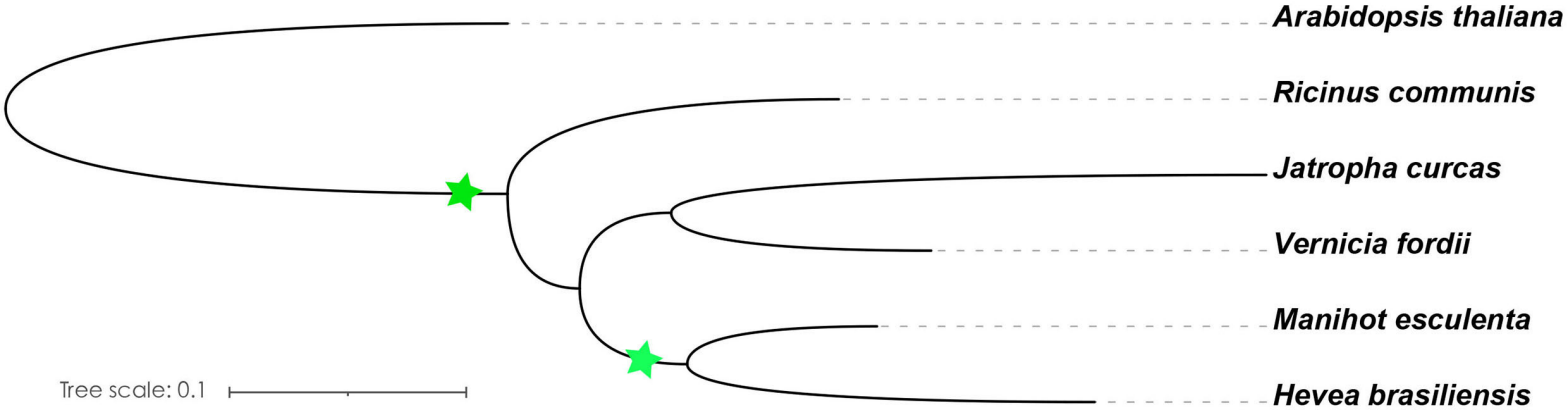
Species evolution tree of *A. thaliana* and Euphorbiaceae species. The OrthoFinder was used to investigate the single-copy orthologs (Emms and Kelly, 2019), and IQ-TREE was used to construct a species evolution tree (Nguyen et al., 2015). The green star represents the WGD event.

Light is one of the most important environmental regulators that affect the flowering in plants (Li et al., 2016). The photoperiod pathway is the main way for plants to monitor the light environment to perceive the time of day and season (Searle and Coupland, 2004). The number of identified *A. thaliana* flowering-related genes participating in the photoperiod pathway is 65, only 47 of which contain orthologs in the Euphorbiaceae genomes. *R. communis*, *V. fordii*, and *J. curcas* contain 52, 56, and 47 genes, respectively, which are part of the photoperiod pathway. We also identified 53 genes in *H. brasiliensis* and 82 genes in *M. esculenta* as putative orthologs of these *A. thaliana* flowering genes (**Table 1**). A recent whole-genome duplication episode that occurred in both *M. esculenta* and *H. brasiliensis* (**Figure 2**), but not in *R. communis*, *V. fordii*, and *J. curcas*, probably played key roles in the expansion of flowering-related genes in *M. esculenta* (Chan et al., 2010; Wang W. et al., 2014; Ha et al., 2019; Zhang et al., 2019b; Liu J. et al., 2020). However, these genes of *H. brasiliensis* have experienced a gene loss event after a recent whole-genome duplication (WGD) event, resulting in its number less than *M. esculenta*. Among 65 genes involved in the photoperiod pathway in *A. thaliana*, 18 genes were not found to have orthologs in the five Euphorbiaceae genomes.

In *A. thaliana*, the *PHYTOCHROME*(*PHY*)*A*, *PHYB*, *PHYC*, *PHYD*, and *PHYE* genes encoded phytochrome proteins that perceived the red and far-red light (Clack et al., 1994). Additionally, *PHYA* is unique because it can be activated by light and can be degraded specifically (Weller et al., 2004; Debrieux and Fankhauser, 2010). Three, five, three, three, and three orthologous genes of *A. thaliana PHY* genes were detected in *R. communis*, *V. fordii*, *J. curcas*, *M. esculenta*, and *H. brasiliensis*, respectively. *CRYPTOCHROME 1* (*CRY1*) and *CRY2* encoded cryptochrome proteins which are sensitive to blue light (Ahmad et al., 1998; Holtkotte et al., 2017). The *A. thaliana CRY* genes contain two, two, two, two, and three homolog genes in *R. communis*, *V. fordii*, *J. curcas*, *H. brasiliensis*, and *M. esculenta*, respectively, while no homolog of *CRY2* was detected in *H. brasiliensis*. Light is a key signal not only for plant growth and development but also for photosynthetic energy production. The light induction of *circadian clock associated 1* (*CCA1*) and *late elongated hypocotyl* (*LHY*) genes was affected by the multiple events; the complex of PHYB–PIF3 binds promoter regions of these two genes (Imaizumi, 2010). In the study, regardless of the *CCA1*, *LHY*, or *PIF3* gene, we have identified a single-copy orthologous gene in *R. communis*, *V. fordii*, and *J. curcas*, *M. esculenta*, and *H. brasiliensis*.

The central oscillator of the circadian clock was produced by *CCA1* and *LHY* together with *TIMING OF CAB EXPRESSION 1* (*TOC1*) (Alabadıí et al., 2001). *TOC1* encodes a nuclear protein containing a CONSTANS, CO-like, and TOC1 (CCT) motif, and the expression of these genes is regulated by the antagonism of *CCA1* or *LHY* (Alabadıí et al., 2001). *R. communis*, *V. fordii*, *J. curcas*, *H. brasiliensis*, and *M. esculenta* have one, one, one, one, and two orthologous genes of *TOC1*, respectively. Three, two, one, six, and six orthologous genes belonging to the *PRR* family were identified in *R. communis*, *V. fordii*, *J. curcas*, *H. brasiliensis*, and *M. esculenta*, respectively. *LOV KELCH PROTEIN 2* (*LKP2*), *FLAVIN-BINDING KELCH REPEAT F-BOX 1* (*FKF1*), and *ZEITLUPE* (*ZTL*) blue-light photoreceptors contribute to regulate the photoperiodic flowering and circadian clock pathway (Imaizumi et al., 2005; Baudry et al., 2010). These proteins have a LOV (light-oxygen voltage-sensing) domain, repeated Kelch motifs, an F-box, and a PAS (Per-Arnt-Sim) domain (Boss et al., 2004). *GIGANTEA* (*GI*) is co‐expressed with an F‐box gene *FKF1* and binds both to *CYCLING DOF FACTOR 1* (*CDF1*)–*CDF5* and to *GI* under light conditions (Sawa et al., 2007; Song et al., 2012). *CONSTANS* (*CO*) encodes crucial regulators of floral transition, day-length perception, and photoperiodic gene expression, and its expression is directly suppressed by *CYCLING DOF FACTORS* (*CDFs*) (Fornara et al., 2009; Goralogia et al., 2017). The upregulation of *CO* during development marks the timing of flowering, as it causes the accumulation of high *FT* transcripts to the threshold level required to trigger flowering (Valverde et al., 2004; Wang C.-Q. et al., 2014). A schematic of this GI–CO–FT regulatory module in the photoperiod pathway has also been found in other plants and is highly conserved, such as soybean, maize, and rice (Hayama et al., 2003; Zhang et al., 2019a). The *LKP2*, *FKF1*, and/or *ZTL* genes contain 11 orthologous genes among these five Euphorbiaceae genomes. Remarkably, we also noted that the *GI* gene was conserved because *R. communis*, *J. curcas*, *H. brasiliensis*, and *M. esculenta* have a single-copy gene, while this gene contains two orthologous genes in *V. fordii*.

### Vernalization pathway

Cold winter reduces the reproductive success rate of plants growing in temperate regions; therefore, these plants have produced the vernalization pathway during evolution, requiring a period of low temperature before the transition of flora (Sung and Amasino, 2004; Jung and Müller, 2009; Bouché et al., 2017). The vernalization pathway is involved in the transduction process and the signal perception that flowering occurs after winter (Luo and He, 2020; Matar et al., 2020). The previously studies revealed that 32 flowering-related genes of *A. thaliana* played key roles in the vernalization pathway. Among these genes, 18 genes have not been detected in the five Euphorbiaceae genomes. *V. fordii*, *R. communis*, *J. curcas*, and *H. brasiliensis* contain 14, 15, 9, and 11 genes, respectively, which are likely related to the vernalization pathway. In *M. esculenta*, we identified 17 genes to be orthologous genes of these *A. thaliana* genes.

*FLOWERING LOCUS C* (*FLC*) is a key gene for vernalization, which can effectively inhibit flowering (Putterill et al., 2004; Deng et al., 2011; Madrid et al., 2020). The repressor of *FLC* is the *REDUCED VERNALIZATION RESPONSE 1* (*VRN1*) and *VRN2* (Putterill et al., 2004). Among them, *VRN1* encodes a transcription factor that contains a B3 DNA-binding domain, and *VRN2* encodes a protein with a zinc-finger motif belonging to polycomb group (PcG) proteins (Gendall et al., 2001; Zhou et al., 2019). Both *VRN1* and *VRN2* can affect the expression of *FLC*, indicating that epigenetic changes in chromatin structure at the locus of *FLC* are a molecular machinery basis for this cellular vernalization memory (Jean Finnegan et al., 2005; Whittaker and Dean, 2017). *A. thaliana VRN1* was conserved in *R. communis*, *V. fordii*, *J. curcas*, and *M. esculenta* containing a single-copy gene. *V. fordii* and *M. esculenta* each contain a single-copy *VRN2* gene, and two and two orthologous genes were found in *R. communis* and *H. brasiliensis*, respectively. Recently, some researchers have found some equivalents of the *A. thaliana FLC* regulators, such as *VERNALIZATION INDEPENDENCE 3* and *4* (*VIP3* and *VIP4*), *PHOTOPERIOD-INDEPENDENT EARLY FLOWERING 1* (*PIE1*), *PHOTOPERIOD-EARLY FLOWERING IN SHORT DAYS* (*EFS*), and *EARLY IN SHORT DAYS 4* (*ESD4*). *PIE1*, an imitation switch (ISWI) family member, played important roles in floral repression and *FLC* activation (Noh and Amasino, 2003; Ojolo et al., 2018). *ESD4*, a SUMO-specific protease, is required for *FLC* expression (Jean Finnegan et al., 2005). The FLC chromatin structure depends on EFS containing histone-lysine N-methyltransferase activity. VIP3 contains multiple WD repeats, and VIP4 is a novel protein playing key roles in the PAF1 transcriptional complex (Takagi and Ueguchi, 2012). *R. communis*, *V. fordii*, *J. curcas*, and *M. esculenta* each have a single-copy *PIE1* gene, and two orthologous genes were found in *H. brasiliensis*. Both *EFS* and *ESD4* have one orthologous gene in *R. communis*, *V. fordii*, and *J. curca* and two orthologous genes in *M. esculenta* and *H. brasiliensis*. *A. thaliana VIP3* and *VIP4* were conserved in these five Euphorbiaceae genomes containing a single-copy gene, except for *H. brasiliensis* which possesses two orthologous *VIP4* genes. *A. thaliana PAF1* and *PAF2* were conserved in these five Euphorbiaceae genomes containing a single-copy gene, except for *J. curca* which does not contain any orthologous *PAF2* gene. Also, some researchers have found that there is a vernalization response FLC-independent because flc null mutants contain functions with a vernalization-sensitive phenotype (Michaels and Amasino, 1999; Michaels and Amasino, 2001). After vernalization of the FLC null mutant, the expression of *FT* and *SOC1* was upregulated, suggesting that FLC-independent and -dependent vernalization branches share a common target (Moon et al., 2003). Alexandre and Hennig found that the MADS transcription factor AGAMOUS-LIKE 24 (AGL24) may be a target because vernalization upregulates the expression of AGL24, which provides an FLC-independent pathway for regulating flowering time (Alexandre and Hennig, 2008). There are three *M. esculenta* genes, two *R. communis* genes, two *V. fordii* genes, one *J. curcas* gene, and one *H. brasiliensis* gene identified to be orthologous to *AGL24.*

### Autonomous pathways

Autonomous pathways include posttranscriptional genes and epigenetic regulation, which can control the flowering time in plants (Simpson and Dean, 2002; Simpson, 2004). There are 19 genes involved in the autonomous pathway in *A. thaliana*. *V. fordii*, *R. communis*, *J. curcas*, *M. esculenta*, and *H. brasiliensis* have 19, 20, 18, 26, and 22 genes, respectively, which are part of the autonomous pathway. The functions of the autonomous pathway repress flowering by promoting the accumulation of an mRNA that is a MADS-domain transcription factor, *FLOWERING LOCUS C* (*FLC*) (Michaels and Amasino, 2001). FCA is an RNA-binding protein that can interact with FY to downregulate the expression of FLC (Quesada et al., 2003), thereby promoting flowering. *A. thaliana FCA* was conserved in these five Euphorbiaceae genomes containing a single-copy gene, except for *H. brasiliensis* which does not possess any orthologous *FCA*. *FY* contains one or two orthologous genes in these five Euphorbiaceae genomes. FLOWERING LATE KH MOTIF (FLK), an RNA-binding protein, can regulate the autonomous pathway *via* FLC (Lim et al., 2004). FPA encodes a protein involved in floral induction having RNA-recognition motifs (Schomburg et al., 2001). FVE, a retinoblastoma-related protein with a WD-repeat domain, can bind to chromatin and regulate flowering time (Ausín et al., 2004). *FPA* has one orthologous gene in *R. communis*, *J. curcas*, and *M. esculenta*. FLK contains one or two homologs in these five Euphorbiaceae genomes. The *MSI* family contains four members, such as *FVE*, which have five, six, five, seven, and five orthologs in *V. fordii*, *J. curcas*, *R. communis*, *M. esculenta*, and *H. brasiliensis*, respectively. *LUMINIDEPENDENS* (*LD*) encodes a nuclear protein with a homeodomain, which plays an important role in RNA processing, such as *FPA* and *FCA*. *FLOWERING LOCUS D* (*FLD*) is another autonomous gene that can inhibit the expression of *FLC* to control flowering time. *A. thaliana FLD* and *LD* were conserved in these five Euphorbiaceae genomes with a single-copy gene, except for *H. brasiliensis* which does not contain any orthologous *FLD* gene and contains two orthologous *LD* genes.

### Floral pathway integrator

The signaling pathways that transmit and receive input signals include the autonomous pathway, the ambient temperature, the vernalization, and the photoperiod pathways (Andrés and Coupland, 2012). Floral pathway integrator genes can integrate the input from these pathways (Simpson and Dean, 2002; Van Dijk and Molenaar, 2017). Although there are multiple pathways with associated genes involved in the regulation of flowering, the expression level of *FT* largely determines the flowering time. *FT*, *SOC1*, and *LFY* integrate multiple pathways and then make a single decision of developmental (Moon et al., 2005). In *A. thaliana*, 29 genes were identified to classify as FPIs. *V. fordii*, *J. curcas*, *R. communis*, *M. esculenta*, and *H. brasiliensis* contain 19, 16, 24, 34, and 23 genes, respectively, which are putatively associated with FPIs.

The transcriptional activation of *FT*, an activator of flowering, can be induced by the activation of the photoperiod flowering pathway (Steinbach and Hennig, 2014). The function of *FT* is mainly as a mobile flowering signal, which is generated in the leaves and then transferred to the shoot apical meristem (SAM) (Turck et al., 2008). In SAM, FT interacts with FD to produce an FT–FD complex and then activates other FPI genes, such as *LFY* and *SOC1* (Kaneko-Suzuki et al., 2018; Li et al., 2019). In the study, we identified one, one, five, three, and one gene in *V. fordii*, *J. curcas*, *R. communis*, *M. esculenta*, and *H. brasiliensis* to be orthologous to *FT* and its homolog *TWIN SISTER OF FT* (*TSF*), respectively. *FT* and floral repressor terminal flower 1 (*TFL1*), belonging to the same *Raf* family, contain antagonistic functions. Each *V. fordii*, *J. curcas*, *R. communis*, and *M. esculenta* possess one ortholog of *TFL1*. *SOC1* and *LFY* genes play vital roles in the regulation of the flowering network. *SOC1* can link floral development and floral induction by regulating the expression of *LFY*. Each *R. communis*, *H. brasiliensis*, and *M. esculenta* contain two orthologs of *SOC1*, and the remaining two species contain one *SOC1* ortholog. *LFY* contains one orthologous gene in *R. communis*, *J. curcas*, and *V. fordii*, while *LFY* contains two orthologs in *M. esculenta* and *H. brasiliensis*. In this study, we also considered *GENERAL REGULATORY FACTOR* (*GRF*) and *NUCLEAR FACTOR Y* (*NF-Y*) due to these two transcription factors that have been confirmed to be involved in flower development. For *NF-Y* genes encoding the basic helix–loop–helix ID factors, *V. fordii*, *J. curcas*, *R. communis*, *H. brasiliensis*, and *M. esculenta* have three, seven, nine, six, and 11 orthologs, respectively. There are 11 genes of the *GRF* family containing 12, 6, 10, 12, and 17 homologs of *V. fordii*, *J. curcas*, *R. communis*, *H. brasiliensis*, and *M. esculenta*, respectively.

### Ambient temperature pathway

The biomass and architecture of plants can be dramatically affected by changes in ambient temperature (Wigge, 2013). The global temperatures seem to be rising, so understanding how plants respond to changes in ambient temperatures can help plants adapt to different climatic conditions. In response to changes in ambient temperatures, plants can make corresponding measures to control flowering time. The floral integrator *FT* can be activated independently of *CO* expression at high temperatures and seems to partially mediate the ambient temperature pathway (Wigge, 2011). *SHORT VEGETATIVE PHASE* (*SVP*), a MADS-box gene, can negatively regulate the expression of *FT* by directly binding to the *FT* sequence (Lee et al., 2013). FLC interacts with SVP to generate a complex to control the flowering time (Li et al., 2008). We also found genes homologous to *SVP* in these five Euphorbiaceae genomes. The *FVE* and *FCA* in the autonomous pathway have been confirmed to take part in the perception of ambient temperatures affecting flowering time. Different ambient temperatures can activate different photoreceptors. For example, *PHYE* is a contributor to the main phytochrome at 16°C. The *PSEUDO-RESPONSE REGULATOR 7* (*PRR7*) and *PRR9* genes expressed in the morning contain dual functions in the circadian rhythm, participating in the regulation of the central oscillator and the transmission of light signals to the clock (Farré et al., 2005). The MADS-box gene, *FLOWERING LOCUS M* (*FLM*), can inhibit flowering in response to temperature. At ambient temperature, *EARLY FLOWERING 3* (*ELF3*) and *TFL1* have complementary effects on regulating flowering time (Strasser et al., 2009). Actually, Arabidopsis thermosensitive flowering may require genes from many different pathways, which are involved in the control of *FT* expression.

### Gibberellin pathway

Among various phytohormones, gibberellin can induce flower formation and promote flowering in the model plant *A. thaliana* (Fornara et al., 2010). The GA pathway acts by inducing *LFY*, *SOC1*, *FT*, etc. The *O-linked N-acetylglucosamine transferase SPINDLY* (*SPY*) acts as a negative regulator to regulate the gibberellin (GA) signaling pathway (Jacobsen and Olszewski, 1993). This gene contains two orthologs in *M. esculenta* and one in each of the remaining four Euphorbiaceae genomes.

### Floral meristem identity genes

The meristem identity genes can be classified as the shoot meristem identity genes and the floral meristem identity genes, and the latter genes are necessary for the developing floral primordia (Grandi et al., 2012). In the initial stage of flower development, flower meristems will not form organs, but their size will increase to a certain extent. Among these genes, the functions of *CAULIFLOWER* (*CAL*), *FRUITFUL* (*FUL*), *APETALA 1* (*AP1*), *UNUSUAL FLORAL ORGANS* (*UFO*), and *LFY* can promote floral meristem identity. *LFY*, a meristem identity gene, is a key player in flower development (Silva et al., 2016). Three genes, namely, *SHOOTMERISTEMLESS* (*STM)*, *WUSCHEL* (*WUS*), and *TFL1*, play key roles in maintaining the identity of inflorescence shoot meristems (Sablowski, 2007; Kim M. Y. et al., 2013). Some genes, such as *PISTILLATA* (*PI*), *AGAMOUS* (*AG*), *APETALA 3* (*AP3*), and *AP2*, have also been examined because these genes may be involved in mediating floral organ identity and meristem function (Riechmann et al., 1996; Fornara et al., 2010). In *A. thaliana*, 25 genes were found to contribute to the development of floral organs and the establishment of floral meristems. Remarkably, five genes, namely, *SEPALLATA3* (*SEP3*), *APETALA 3* (*AP3*), *SEEDSTICK* (*STK*), *ENHANCER OF AG-4 2* (*HUA2*), and *PISTILLATA*, were not examined in any of these five Euphorbiaceae genomes. *V. fordii*, *J. curcas*, *R. communis*, *H. brasiliensis*, and *M. esculenta* contain 20, 20, 18, 15, and 33 orthologs of *A. thaliana* genes, respectively. *APETALA 1* (*AP1*), *CAL*, and *FUL* were paralogs, each encoding a MADS-box domain (Alvarez-Buylla et al., 2006). AP2 belongs to the ethylene-responsive element-binding protein (EREBP) family encoding the AP2 domain that is involved in the control of flower. These data indicate that the transcriptional regulatory network plays an important role in the specification of floral organs and meristems. UFO is an F-box protein that is required for bract suppression and floral-meristem identity (Hepworth et al., 2006). *V. fordii* contains three orthologs of *AP1*, and one ortholog of *AP2*. *R. communis* has two orthologs of *AP1* and one ortholog of *AP2*. One ortholog of *AP1* and two orthologs of *AP2* were found in *H. brasiliensis*. There are four and two orthologs of *AP1* and *AP2* identified in *M. esculenta* separately, while only two orthologs of *AP1* were detected in *J. curcas*. The number of homologs of CAL in *J. curcas*, *V. fordii*, and *R. communis* is two, while *M. esculenta* contains three *CAL* orthologs. The function of *FUL* mainly affects many biological processes including but not limited to controlling cauline leaf morphology, meristem identity, and flowering time. *AG* is required to specify the identity of floral organs in *A. thaliana*. By contributing to meristem and floral organ identity, members of the SEP family are required to specify the “floral state”. *UFO* has been reported to be involved in both floral organ and meristem development (Hepworth et al., 2006). There are multiple *FUL* copies in *R. communis* and *M. esculenta* while *FUL* is conserved having a single-copy gene in *H. brasiliensis*, *J. curcas*, and *V. fordii*. *J. curcas* lack *AG* orthologs, but this gene is conserved in four other genomes containing a single-copy gene. *J. curcas*, *R. communis*, and *H. brasiliensis* have one, two, and two members of the *SEP* family, respectively, while *V. fordii* contains six and *M. esculenta* has five members of the *SEP* family. *UFO* contains one homolog gene in *J. curcas*, while the other four genomes lack *UFO* homologs.

### Comparison of flowering-related genes in *A. thaliana* and Euphorbiaceae genomes

In *A. thaliana*, 207 flowering-related genes were identified to play important roles in the control of flowering time. In this study, we found that 39 genes did not contain orthologous counterparts among these five Euphorbiaceae genomes, such as FLC, *CO-LIKE* (*COL*), *FRI*, and *MAF* (**Tables S1** and **Table S2**). The lack of some flowering-related genes orthologs has been confirmed by previously published papers (Kim M. Y. et al., 2013). For example, Kim found that *Lotus corniculatus*, *Glycine max*, and *Medicago truncatula* lack 56 orthologous of *A. thaliana* flowering-related genes, such as *CO* and *ELF4*, indicating that the photoperiod pathway is regulated in a *CO*-independent manner in legume species (Kim M. Y. et al., 2013). In our study, *R. communis*, *V. fordii*, and *H. brasiliensis* lack *CO* orthologs, and *J. curcas* and *M. esculenta* contain two *CO* orthologs, suggesting that different plants have evolved different ways for activating the photoperiod pathway. The transcriptional activation of *FT* is achieved by *CO* directly binding its promoter, which may recruit different microRNAs or DNA-binding proteins to medicate *FT* expression (Fujiwara et al., 2008). This way has been mentioned for the photoperiod but the CO-independent pathway to control flowering time by regulating the expression of *FT*. Indeed, some researchers have found a CO-independent pathway to regulate the expression of *FT* which is directly activated by miR172 or GI (Jung et al., 2007; Sawa and Kay, 2011).

As central players in the autonomous pathway and vernalization pathway, both *FLC* and *FRI* genes were involved in regulating the flowering time in *A. thaliana* (Fornara et al., 2010). In our study, we did not detect the homologs of *FRI* and *FLC*, as well as the *MAFs* (FLC homologs). This phenomenon has been found in several previous studies. The FLC-FRI module mechanistically interacts to prevent *A. thaliana* from flowering before vernalization (Townsend et al., 2018). Silencing *FLC* genes to regulate flowering time is one of the most typical examples of the role of chromatin remodeling and non-coding RNA in epigenetic control (Wigge, 2011). In germplasms that require long-term vernalization, *FLC* expression is reactivated after unsaturated vernalization, but this reactivation gradually weakens with increasing cold exposure (Nishio et al., 2020). In our study, the FLC locus was not found in vernalization-non-responsive *R. communis*, *V. fordii*, *H. brasiliensis*, *J. curcas*, and *M. esculenta*, which were consistent with their characteristics. The deletion of some key genes in the flowering pathway may help to solve other problems related to molecular circuits in the flowering pathway.

### Distribution of flowering-related genes and putative gene regulatory network in the Euphorbiaceae genomes

In this study, we distributed all orthologs of flowering-related genes in *A. thaliana* throughout the genomes of *V. fordii*, *J. curcas*, *R. communis*, *H. brasiliensis*, and *M. esculenta*. There are similar numbers of flowering-related orthologs in *V. fordii*, *J. curcas*, *R. communis*, and *H. brasiliensis*. Comparisons among paralogous orthologous genes of these five Euphorbiaceae genomes suggest that an ancient WGD was shared by these five genomes, and a recent WGD occurred prior to the split of the *H. brasiliensis* and *M. esculenta* (Chan et al., 2010; Wang W. et al., 2014; Ha et al., 2019; Zhang et al., 2019b; Liu J. et al., 2020). We noted that *H. brasiliensis* has also experienced two WGDs, but there are similar numbers of flowering-related orthologs in *V. fordii*, *J. curcas*, *R. communis*, and *H. brasiliensis*. However, the number of flowering-related genes contained in *M. esculenta* is much greater than the other four Euphorbiaceae genomes. These data may reflect a recent WGD which appears to have produced about two times more homologs genes than are found in *V. fordii*, *J. curcas*, and *R. communis* (**Figure 2**). However, the number of flowering-related genes in *H. brasiliensis* may have experienced a gene loss event after two WGDs, which ultimately resulted in the number of genes being basically the same as the other three Euphorbiae species. In the study, although we found that some number of flowering-related genes have multiple copies in these Euphorbiaceae genomes, several flowering-related genes still exist and remain in the form of single copies (**Figure 1**). In *V. fordii*, *J. curcas*, and *R. communis*, 123, 100, and 120 genes of *A. thaliana* are conserved in the form of single copies, respectively, while both of *H. brasiliensis* and *M. esculenta* contain 61 single copies. Totally, nine common genes were identified as conserved single-copy genes among these five Euphorbiaceae species (**Figure 1**).

**Figure 3:**
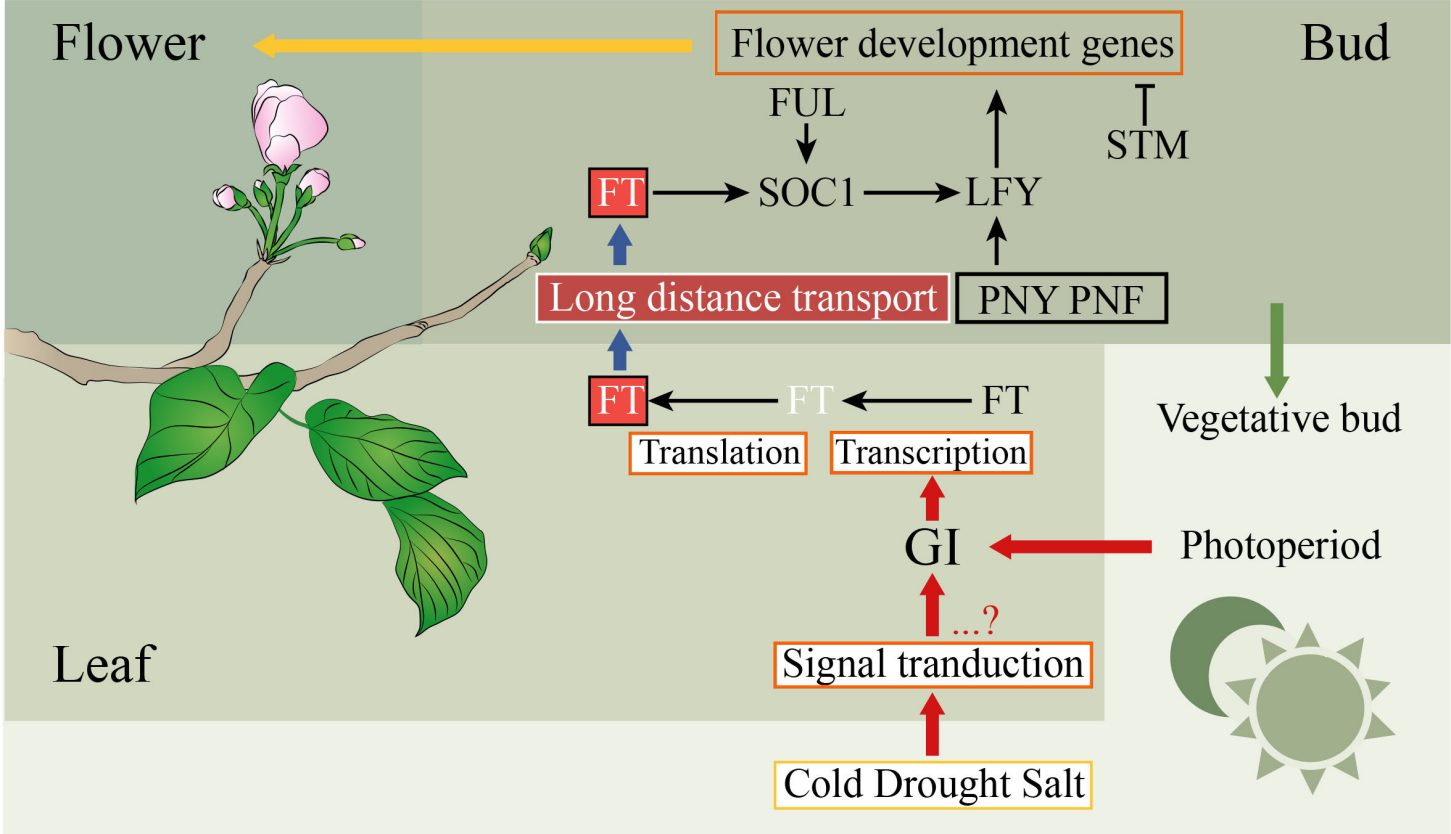
Simplified pathways to participate in the regulation of flowering in plants. The positive and negative controls are represented by arrows and perpendicular lines, respectively. The physiological processes, mRNA, genes, and proteins are represented by orange frames, white letters without frames, black letters without frames, and other frames, respectively.

The probability of blooming costs is related to the success rate of reproduction, which may have led to plants evolving to develop a set of metabolic and genetic mechanisms to sense and respond to changes in their own environment (Liu Y. et al., 2020). The regulatory network of the SAM destiny is complex, which depends on multiple exogenous and endogenous factors (Liu Y. et al., 2020). In recent decades, both complex networks and mechanisms of flowering have been well studied in the model plant *A. thaliana* (Fornara et al., 2010). However, few studies of flowering have been performed in the Euphorbiaceae species. Based on the above results, the regulatory network of flowering was inferred in Euphorbiaceae plants (**Figure 3**). *FT* controls the flowering time by converging environmental signals sensed by leaves, such as temperature and photoperiod (Corbesier et al., 2007; Wigge, 2011; Liu Y. et al., 2020). The circadian clock in leaves of the plant can receive day-length information. The circadian clock which included *GI* and *PRR7* can regulate the expression of *FT* and positively control flowering (Farré et al., 2005; Mizoguchi et al., 2005). In the phloem of the leaf, *FT* will be transcribed and translated, and then its proteins move to the shoot apex (Corbesier et al., 2007). The transcription level of the circadian clock gene *GI* is regulated by abiotic stresses such as cold, drought, and salt stress, which were related to floral induction (Riboni et al., 2013; Fornara et al., 2015). Many physiological processes including fruit ripening, seed germination, flowering, and growth require *GA* to participate. DELLA protein, a negative regulator of GA signaling, belongs to the GRAS family (Yoshida et al., 2014). In the SAM, the expression of the FMI identity gene *AP1* can be directly regulated by *FT*, while the control of *LFY* requires *FT* to activate *SOC1* (Lee and Lee, 2010). In plants, there is an FT-SOC1-LFY model to activate *LFY* to induce flowering. In our study, the homologous genes of these *FT*, *SOC1*, and *LFY* were identified in these Euphorbiaceae genomes, indicating that this model played a major role in these species.

## Conclusion

In the study, the orthologous counterparts of *A. thaliana* flowering-related genes were identified in five Euphorbiaceae species, *V. fordii*, *J. curcas*, *R. communis*, *M. esculenta*, and *H. brasiliensis*. Most *A. thaliana* flowering-related genes have been detected in Euphorbiaceae genomes, suggesting that basic flowering pathways may be relatively conservative in different plants during evolution. These data will provide new perspectives and potential candidate genes to regulate the timing of flowering on molecular processes in Euphorbiaceae, which have important economic values.

## Data availability statement

The original contributions presented in the study are included in the article/**Supplementary Material**. Further inquiries can be directed to the corresponding authors.

## Author contributions

LJ designed this research and then wrote the manuscript. LJ, TF and JX participated in the evaluation of the manuscript revision. LW and JX prepared the figures and tables. LW, LZ and JX contributed to the provided guidance of the whole study. TF and JX reviewed the manuscript. All authors contributed to the article and approved the submitted version.

## Funding

This work was supported by the Natural Science Fund Project of Hunan Province (Grant No. 2021JJ41068 and 2020JJ4049) and the Outstanding Youth of the Education Department of Hunan Province (Grant No. 20B617).

## Conflict of interest

The authors declare that the research was conducted in the absence of any commercial or financial relationships that could be construed as a potential conflict of interest.

## Publisher’s note

All claims expressed in this article are solely those of the authors and do not necessarily represent those of their affiliated organizations, or those of the publisher, the editors and the reviewers. Any product that may be evaluated in this article, or claim that may be made by its manufacturer, is not guaranteed or endorsed by the publisher.

## Supplementary material

Supplementary Table 1 The list of homologys of the *A. thaliana* flowering‐related genes in Euphorbiaceae species, including castor bean (*Ricinus communis*), physic nut (*Jatropha curcas*), tung tree (*Vernicia fordii*), cassava (*Manihot esculenta*), and rubber tree (*Hevea brasiliensis*).

Supplementary Table 2 Lack of homology of the *A. thaliana* flowering‐related genes in Euphorbiaceae species, including castor bean (*Ricinus communis*), physic nut (*Jatropha curcas*), tung tree (*Vernicia fordii*), cassava (*Manihot esculenta*), and rubber tree (*Hevea brasiliensis*).
